# Phenological changes in bamboo carbohydrates explain the preference for culm over leaves by giant pandas (*Ailuropoda melanoleuca*) during spring

**DOI:** 10.1371/journal.pone.0177582

**Published:** 2017-06-14

**Authors:** Katrina K. Knott, Amelia L. Christian, Josephine F. Falcone, Carrie K. Vance, Laura L. Bauer, George C. Fahey, Andrew J. Kouba

**Affiliations:** 1 Conservation and Research Department, Memphis Zoological Society, Memphis, Tennessee, United States of America; 2 Department of Animal Sciences, Texas A&M University, College Station, Texas, United States of America; 3 Biochemistry, Molecular Biology, Entomology, and Plant Pathology Department, Mississippi State University, Mississippi State, Mississippi, United States of America; 4 Department of Animal Sciences, University of Illinois, Urbana, Illinois, United States of America; 5 Department of Wildlife, Fisheries and Aquaculture, Mississippi State University, Mississippi State, Mississippi, United States of America; Sichuan University, CHINA

## Abstract

Seasonal changes in the foodscape force herbivores to select different plant species or plant parts to meet nutritional requirements. We examined whether the search for calorie-rich carbohydrates explained giant panda’s selection for bamboo culm over leaves during spring. Leaves and culms were collected from four *Phyllostachys* bamboos (*P*. *aurea*, *P*. *aureosulcata*, *P*. *glauca*, and *P*. *nuda*) once per month over 18–27 months. Monthly changes in annual plant part nutrients were examined, and compared to seasonal foraging behaviors of captive giant pandas. Although total fiber was greater (p<0.0001) in culm (85.6 ± 0.5%) than leaves (55.3 ± 0.4%) throughout the year, culm fiber was at its lowest in spring (79–85%) when culm selection by giant pandas exceeded 70% of their overall diet. Culm starch also was greatest (p = 0.044) during spring (5.5 ± 1.1%) and 2.5-fold the percentage of starch in leaves (2.2 ± 0.6%). The free sugars in spring culm consisted of a high proportion of glucose (35%) and fructose (47%), whereas sucrose made up 42% of the total free sugar content of spring leaves. Bound sugars in culm consisted of 60% glucose and 38% xylose likely representative of hemicellulose. The concentrations of bound sugars (hemicelluloses) in spring culms (543.7 ± 13.0 mg/g) was greater (p<0.001) than in leaves (373.0 ± 14.8 mg/g). These data help explain a long-standing question in giant panda foraging ecology: why consume the plant part with the lowest protein and fat during the energetically intensive spring breeding season? Giant pandas likely prefer spring culm that contains abundant mono- and polysaccharides made more bioavailable as a result of reduced fiber content. These data suggest that phenological changes in bamboo plant part nutrition drive foraging decisions by giant pandas.

## Introduction

Animals respond to seasonal changes in the environment, and distinguish between feeding patches on the basis of quantity or quality of food. These foraging decisions impact time budgets, nutritional status, and fitness [1, 2]. Organisms confined to a narrow feeding niche have little flexibility when making foraging choices. Within their habitat, they must constantly gauge their environment to ensure that they are selecting the most energy-rich diet items that meet their physiological requirements. Studies of foraging behavior in captive animals allow for the development of a framework that can be applied to free-ranging individuals, and provide insights into the physiological and behavioral adaptations of organisms to their nutritional niche [3].

The giant panda (*Ailuropoda melanoleuca*) is an obligate herbivore with a diet consisting of primarily fibrous bamboo (~99%). Although the giant panda exhibits morphological and physiological adaptations that allow it to process this woody plant for its diet, the giant panda retained the relatively short gastrointestinal tract (GIT) of a carnivore and a rapid digesta passage rate of 6–12 h [4, 5]. The giant panda’s GIT also lacks specialized compartments, such as a rumen or cecum, required for extensive fermentation; hence, their ability to digest and utilize nutrients from bamboo is considerably reduced (dry matter digestibility ranges from 6.9 to 19.6%) [4, 6, 7]. Dierenfeld [8] suggested that the energy requirements of the giant panda ranged from 3,100 to 5,700 kcal/d. Since there is minimal protein and fat content accessible from their diet, giant pandas must consume large quantities of bamboo to meet their daily energy requirements (6–15% of their body weight, ranging 10–30 kg fresh bamboo daily) and spend 50–60% of their time foraging [4, 6, 9, 10].

Phenological changes in bamboo nutrients that differ among habitats, and with environmental conditions and season likely contribute to dietary selection patterns of giant pandas. In late spring and early fall, giant pandas in the Qionglia and Qinling Mountains of China undergo altitudinal migrations between winter and summer habitat ranges [11, 12]. Bamboo species in these mountains are exclusive to particular elevations, and therefore seasonal movements of the giant panda are accompanied by a dietary staple species shift [9]. For example, in the Qionglai Mountains of Wolong Nature Reserve, giant pandas shifted between a winter diet of *Fargesia robusta* and a summer diet of *Bashania fangiana*, whereas giant pandas in the Qinling Mountains of the Foping Nature Reserve shifted between a winter diet of *Bashania fargesii* and a summer diet of *Fargesia qinlingensis* [12, 13]. In addition to consuming alternating bamboo species, giant pandas also can display seasonal preferences in plant parts switching between leaf, culm (stalk), and shoot. For much of the year, giant pandas feed primarily on the leaves of bamboo. However, giant pandas in some sub-populations have been reported to consume a large proportion of culm during the late winter/early spring months and switch back to leaves in mid- to late June [9, 11, 14, 15]. During the bamboo growth season in spring, shoots are eaten almost exclusively. Why giant pandas would select culm, which is significantly higher in fiber content, lower in protein and fat, and less digestible compared to leaves has remained a mystery. The consumption of culm typically precedes or coincides with the breeding season, when giant pandas expend a large amount of energy when seeking mates and engage in physical competitions for breeding rights. These seasonal shifts in home range, feeding site, and preference of bamboo species and plant part indicate that the giant panda is making foraging decisions based on principal components (e.g., nutritional and energetic value) while overcoming extrinsic (e.g., topography) and intrinsic limitations (e.g., metabolic demands).

Although changes in foraging behavior for captive and wild giant pandas have been described to be a response to changes in bamboo nutrient composition [9, 11, 15, 16], the nutrient, or combination of nutrients, driving species and plant part selection by giant pandas is largely unknown. At the Memphis Zoo, giant pandas exhibited a dietary preference for culm during spring followed by a dietary shift to greater leaf consumption in mid-summer that was similar to many of their wild counterparts [15, 16]. We hypothesized that this preference for culm by giant pandas during spring serves a nutritional advantage that allows giant pandas to meet their energy requirements of breeding. One such energy-rich nutrient are carbohydrates that occur in plant tissues as monosaccharides, disaccharides, and polysaccharides and provide energy and structural support for grasses [17]. Carbohydrates have been considered a key energy source for grazing and browsing animals, as starches and free sugars are easily digested and absorbed in both ruminant and non-ruminant GIT [17]. Therefore, we examined annual variations in culm and leaf carbohydrates in relation to the seasonal foraging behaviors of giant pandas. This study provides new insight into the unique foraging behavior of giant pandas, and helps to explain how an obligate herbivore with a carnivore digestive tract can meet its nutritional demands by taking advantage of seasonal plant part-specific calorie fluctuations of its diet.

## Results

### Annual variations in fiber content of bamboo leaves and culm

**Fig 1:**
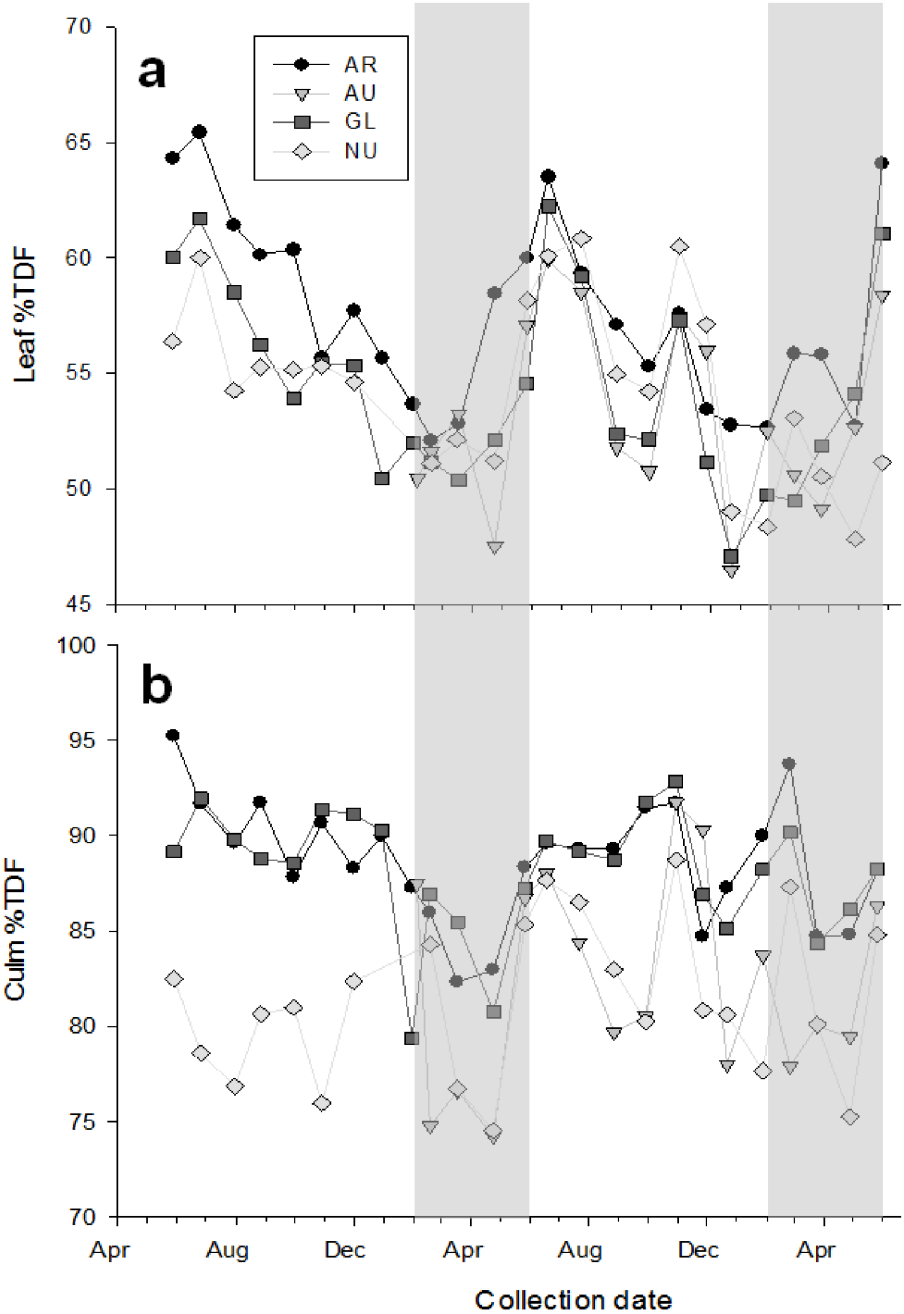
Fiber content in bamboo leaves and culm. Total dietary fiber (TDF) concentrations for (panel a) leaf and (panel b) culm of bamboo species *Phyllostachys* (*P*.) *aurea* (AR), *P*. *aureosulcata* (AU), *P*. *glauca* (GL), and *P*. *nuda* (NU). Samples were collected monthly over a period of 18–27 mo. Data are expressed as a percentage of dry matter (DM). The shaded area represents the time when captive giant pandas consumed primarily culm.

Overall, leaves contained a lower (p<0.001) percentage of total dietary fiber (TDF), acid detergent fiber (ADF), and acid detergent lignin (ADL) than culm throughout the year. All four *Phyllostachys* species followed a similar pattern in leaf TDF over the study period (Fig 1). The lowest TDF content in leaves occurred during February-May with an increase during May and June. Leaf TDF was greatest for all species during July (combined species average, 58.0 ± 0.7%), and declined during the fall and winter months.

Culm TDF remained elevated (p<0.001) above the TDF of leaves regardless of time of year (Fig 1). The combined species average for TDF in culm, however, declined to its lowest value (81.8 ± 1.5%) during culm-eating months in spring (March-May), which was significantly lower (p = 0.009) from culm TDF during other months of the year (Fig 1). The TDF content of culm for all species was highest during the summer months (combined species average, 87.0 ± 0.9%). Culm from AU and NU species had significantly lower (p<0.001) TDF content during June–January (82.7 ± 1.3%) and April–May (85.8 ± 1.0%) than in comparison to AR and GL during these same time periods (June–January, 89.4 ± 0.5%; April and May, 87.5 ± 0.8%).

### Annual variations in protein, lipid, and ash in bamboo leaves and culm

Crude protein was over 4-fold greater (p<0.0001) in leaves than culm (Table 1). The percentage of crude protein in both leaves and culm remained relatively constant throughout the year and differed by less than 2% across individual months. The percentage of EE (fat) and ash was also greater (p<0.001) in leaves than culm (Table 1). The lowest percentage of ash in leaves occurred in May (7.6 ± 0.3% of DM) with a steady increase during June/ July. Maximum ash content for all *Phyllostachys* species occurred during August to February (11.2 ± 0.4%).

**Table 1:** Crude protein (CP), ash, and ether extractable fat (EE) in leaf and culm for four *Phylostachys* bamboo species. *P*. *aurea* (AR), *P*. *aureosulcata* (AU), *P*. *glauca* (GL), and *P*. *nuda* (NU).

Species	Plant Part	CP%	Ash%	EE%
AR	Leaf	17.3 ± 1.9 (13.6–20.3)	9.8 ± 2.1 (6.9–15.5)	1.9 ± 0.5 (1.1–3.3)
	Culm	2.4 ± 0.7 (1.5–3.9)	1.3 ± 0.2 (1.0–1.7)	0.6 ± 0.2 (0.1–1.2)
AU	Leaf	18.0 ± 1.6 (15.4–20.2)	11.3 ± 2.6 (7.1–16.6)	1.8 ± 0.5 (1.1–2.3)
	Culm	3.9 ± 1.2 (1.8–5.7)	1.7 ± 0.5 (1.2–3.0)	0.7 ± 0.4 (0.3–1.8)
GL	Leaf	19.5 ± 1.7 (16.2–24.3)	11.2 ± 2.4 (6.4–16.3)	1.6 ± 0.5 (0.8–3.6)
	Culm	3.9 ± 2.7 (1.8–6.7)	1.9 ± 1.3 (1.3–3.5)	0.6 ± 0.3 (0.2–1.3)
NU	Leaf	17.9 ± 1.8 (14.4–21.6	11.0 ± 2.5 (7.8–17.1)	2.2 ± 0.6 (1.0–2.9)
	Culm	5.0 ± 2.6 (2.3–12.9)	1.9 ± 0.5 (1.4–3.5)	0.6 ± 0.3 (0.2–1.6)

Data are shown as mean ± SEM, with the range shown in brackets for the entire 18–27 sampling period. Data are expressed as percentage of dry matter (DM).

### Annual variations in carbohydrates in bamboo leaf and culm

**Fig 2:**
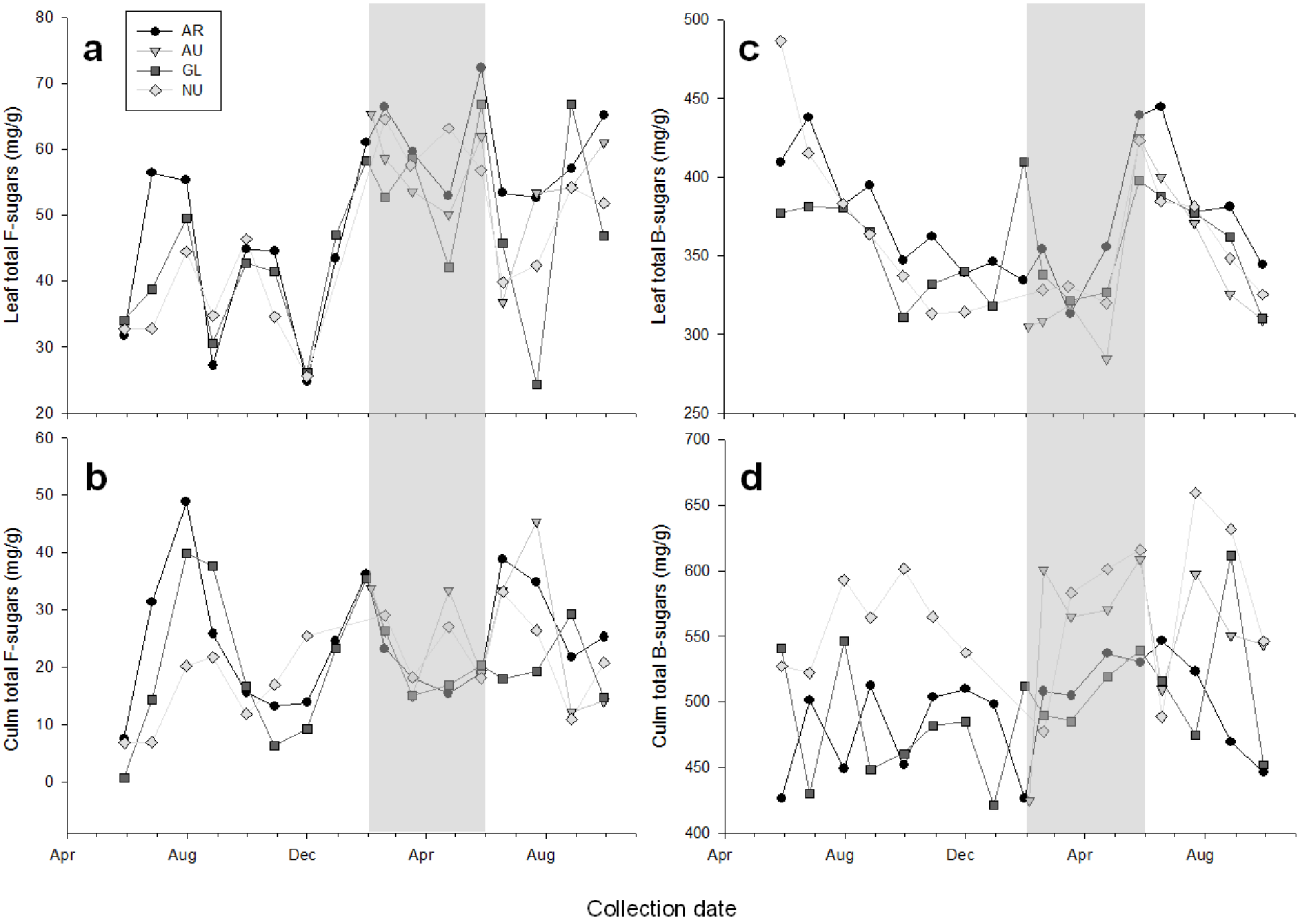
Sugars in bamboo leaves and culm. Total free sugars (panel a, b) and bound sugars (panel c, d) sugars in leaf and culm of bamboo species *Phyllostachys* (*P*.) *aurea* (AR), *P*. *aureosulcata* (AU), *P*. *glauca* (GL), and *P*. *nuda* (NU) expressed on a dry matter (DM) basis. Samples were collected once per month over a period of 18–27 months. The shaded area represents the time when captive giant pandas consumed primarily culm.

The concentration of total free sugars fluctuated between 30 and 70 mg/g in leaves throughout the year, whereas the total content of free sugars in culm ranged 6 to 50 mg/g (Fig 2). Glucose, fructose, and sucrose were the predominant constituents of the free sugar fractions (98–99% of total free sugar content) in leaves and culm of *P*. *aureosulcata*, a representative bamboo preferred by giant pandas, during all months of the year (Table 2). Although the total free sugar content of culm in spring was lower (p<0.001) than free sugar content in leaves (Fig 1, Table 3), the culm preferred by giant pandas during spring contained a greater proportion of glucose and fructose whereas the free sugars in leaves consisted primarily of sucrose (Table 2). *P*. *aureosulcata* leaves contained a greater proportion of free glucose and fructose than sucrose when giant pandas transitioned back to consumption of leaves during summer and fall (July-September; Table 2)

**Table 2:** Percentage of free and bound sugars in leaf and culm of *P*. *aureosulcata*.

		Plant Part	
Part/ Months Preferred	Individual Sugars	Leaf	Culm
Culm Eating Season/	Free		
(March–May)	Glucose %	29	35
	Fructose %	28	47
	Sucrose %	42	16
	Bound		
	Glucose %	43	60
	Xylose %	10	38
	Arabinose %	6	2
	Galactose %	29	bd
Leaf Eating Season/	Free		
(July-September)	Glucose %	36	26
	Fructose %	34	28
	Sucrose %	28	44
	Bound		
	Glucose %	36	55
	Xylose %	47	43
	Arabinose %	11	2
	Galactose %	6	bd

Individual free and bound sugars in leaf and culm expressed as the average percentage of total free or bound sugars.The percentage of arabinose, galactose, xylose, mannose, fucose, and rhamnose in leaf and culm was <1% of total free sugars. The percentage of bound mannose in leaf and culm was below detection limits. Months were selected based on giant panda preference for culm (March-May) and leaf (July-September). bd = below detection.

**Table 3:** Nutrient components (Mean ± SEM) in leaf and culm during spring (March-May) in four *Phyllostachys* bamboos.

	Plant Part		
Item	Leaf	Culm	Statistic
**TDF %**	54.98 ± 0.96	83.34 ± 0.97	t_52_ = 20.71, p<0.001
**Ash %**	10.91 ± 0.64	1.72 ± 0.12	U = 0.0, p<0.001
**Protein %**	18.47 ± 0.43	4.17 ± 0.38	U = 27.0, p<0.001
**EE %**	1.67 ± 0.10	0.55 ± 0.05	U = 9.0, p<0.001
**Starch %**	2.21 ± 0.57	5.54 ± 1.06	U = 238.0, p = 0.044
**Hemicelluloses %**	22.01 ± 1.38	26.29 ± 0.69	U = 30.0, p = 0.006
**Total free sugars mg/g**	53.12 ± 3.04	16.67 ± 2.03	t_29_ = 9.8, p<0.001
**Total bound sugars mg/g**	373.04 ± 14.82	543.65 ± 12.98	t_29_ = 8.6, p<0.001

Differences were assessed using a T-statistic for normal data and Mann-Whitney U statistic for nonparametric data. TDF = total dietary fiber; EE = ether extractable fat.

**Fig 3:**
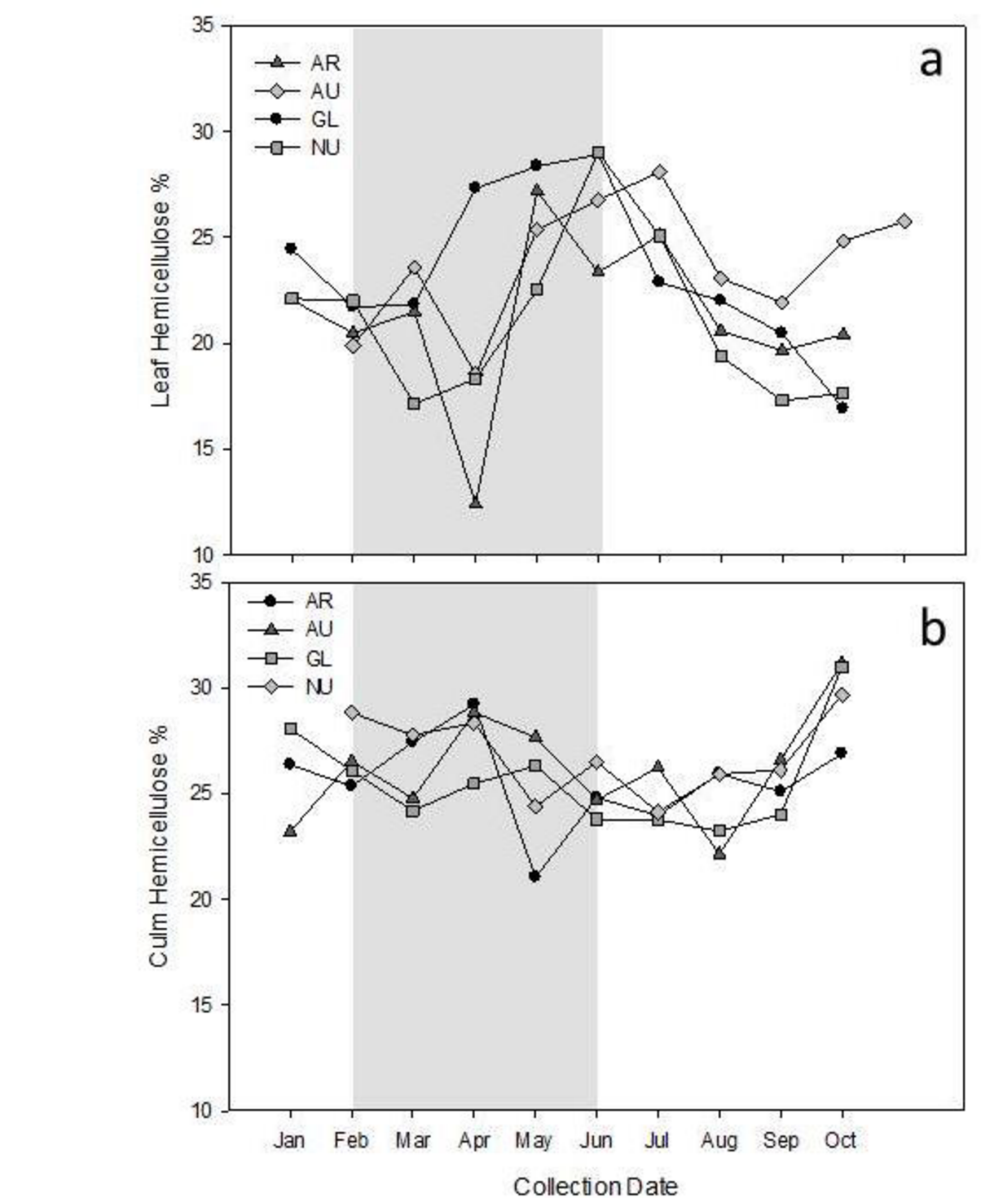
Hemicellulose content of bamboo leaves and culm. Percentage of hemicelluloses in leaf (panel a) and culm (panel b) of bamboo species *Phyllostachys* (*P*.) *aurea* (AR), *P*. *aureosulcata* (AU), *P*. *glauca* (GL), and *P*. *nuda* (NU) expressed on a dry matter (DM) basis. The shaded area represents the time when captive giant pandas consumed primarily culm.

Glucose and xylose were the main constituents of bound sugars for both leaf and culm suggesting that the changing pattern in bound sugars largely represents changes in hemicellulose. This finding was supported by a similar annual pattern of bound sugars in leaf and culm (Fig 2) to the percentage of hemicelluloses in these plant parts (Fig 3). Bound sugars and hemicelluloses in culm remained relatively constant throughout the year, and were greater (p<0.0001) in culm versus leaves during the culm-eating season in spring (Figs 2 and 3, Table 3).Bound sugars and hemicelluloses in leaves were greatest in June (average for all species; bound sugars, 411.1 ± 12.7 mg/g; hemicelluloses, 27.0 ± 1.3%; Figs 2 and 3) when giant pandas transitioned from culm to greater leaf consumption. Bound sugars and hemicelluloses in leaves declined steadily throughout the summer resulting in the lowest values (317.3 ± 15.0 mg/g and 21.0 ± 0.7% of DM, respectively) from late fall through winter and into spring (October-April, Figs 2 and 3).

**Fig 4:**
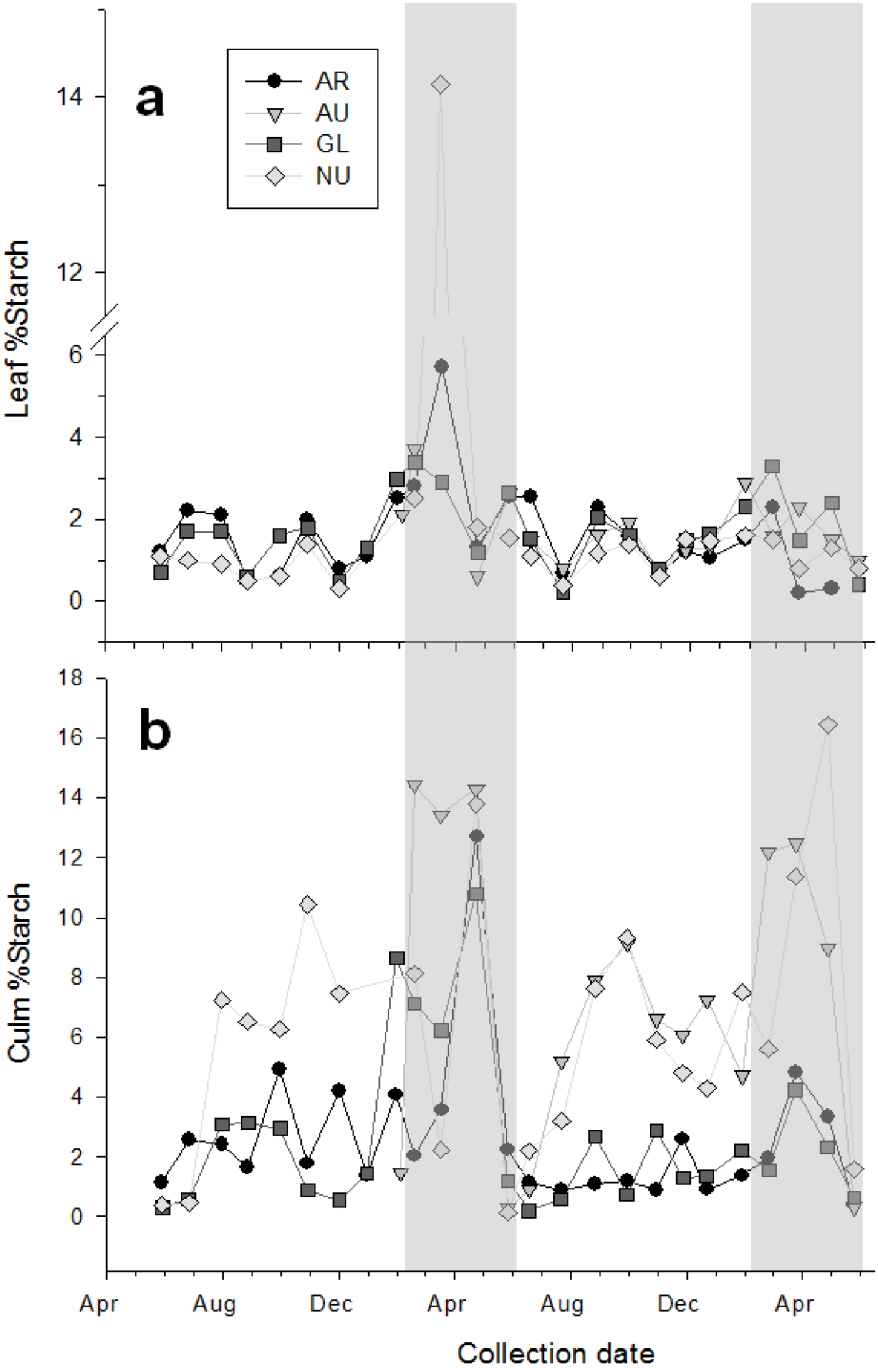
Starch content in bamboo leaves and culm. Starch concentrations for leaf (panel a) and culm (panel b) of bamboo species *Phyllostachys* (*P*.) *aurea* (AR), *P*. *aureosulcata* (AU), *P*. *glauca* (GL), and *P*. *nuda* (NU). Samples were collected once per month over a period of 18–27 mo. Data are expressed as a percentage of dry matter (DM). The shaded area represents the time when captive giant pandas consumed primarily culm.

The percentage of starch in both culm and leaves was greatest during spring (Fig 4), yet starch content in culm was 2.5-fold greater than leaves (Table 3). Starch content in AR and GL culm was similar to leaves during June through December ranging from 0.2 to 5%. However, culm from AU and NU was 2-3-fold higher during this same time (range 0.9–9.3% of DM).

### Phenology of bamboo nutrition in relation to culm selection by giant pandas

**Fig 5:**
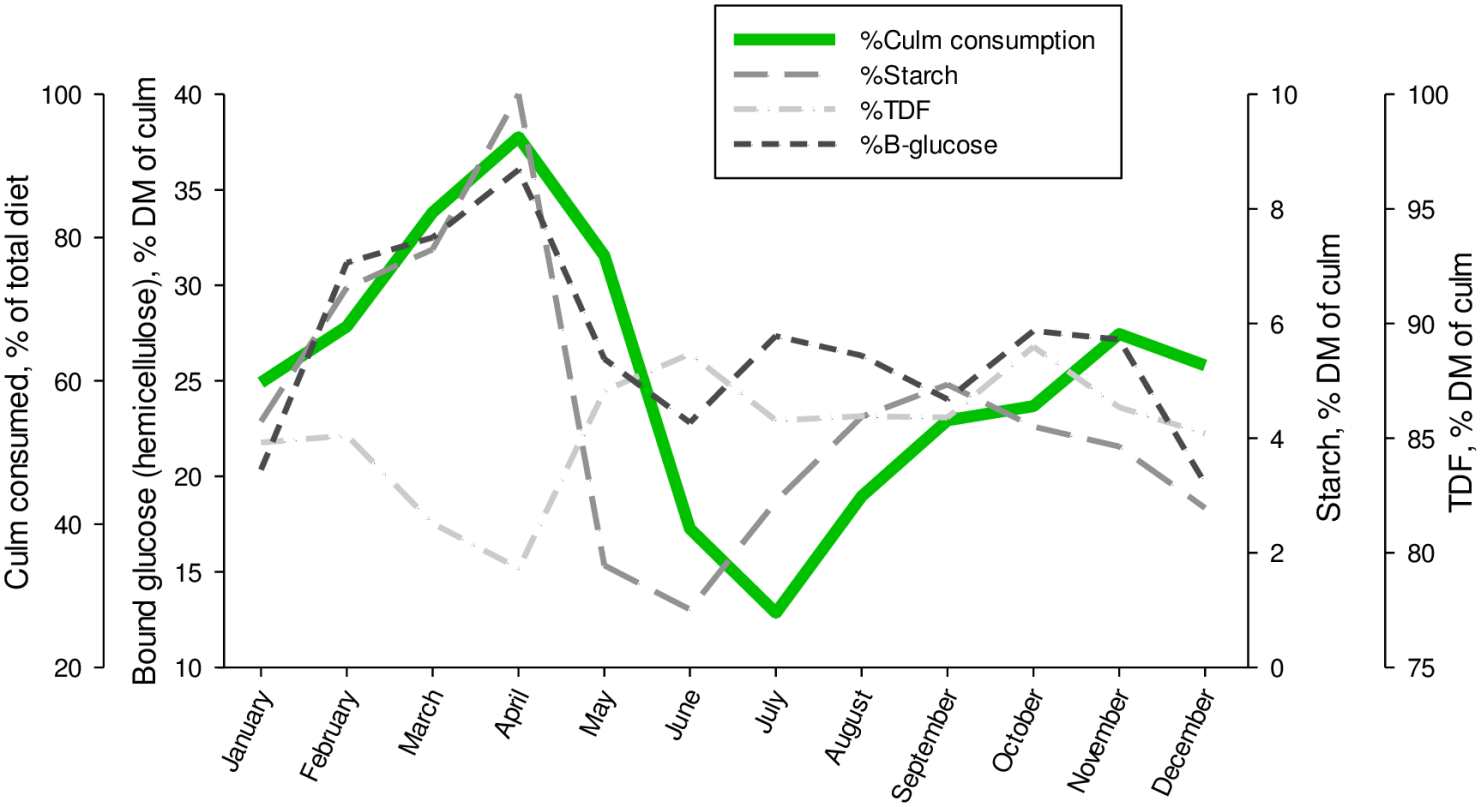
Annual changes in culm nutrients in relation to forage selection by giant pandas. Feeding behavior data for culm consumption were collected at the same time as bamboo were collected for nutritional analyses, and reported as the mean for both animals each month. Bound glucose, starch, and fiber content in culm are shown as the mean of four *Phyllostachys* species during each month of the year. Nutritional data are presented on a dry matter (DM) basis.

Culm consumption by giant pandas was at a minimum during the summer months of June and July and made up less than 40% of the overall diet (Fig 5). Culm consumption increased to 55% of overall diet by September/October and 64% of diet in November/December. By March, culm consumption by giant pandas reached 80% of the total diet and by April, 95% of the diet consisted of culm. Culm selection patterns most mirrored the phenological changes in culm starch and bound glucose. Culm starch (soluble free glucose) was at a minimum (~1% DM) in June and increased to a mean of 5% by September. This increase in culm starch preceded the dietary selection for culm by ~1 month. An additional increase in culm starch occurred from January through March, which was accompanied by a similar increase in the percentage of bound glucose, likely representative of an increase in culm hemicelluloses. By April, both starch (10% of DM) and bound glucose (36% of DM) had peaked in the same month that culm consumption had peaked and culm fiber had reached an overall low. A rapid decline in culm starch and bound glucose occurred during May and preceded the observed decline in the consumption of culm by giant pandas as they transitioned to a greater leaf intake in June.

## Discussion

According to theories of optimal foraging strategy, animals select for diets that contain more calories and select against diet items with relatively high indigestible material, such as fiber [1]. In this study, 80 to 95% of the total spring diet (March-May) of the giant pandas, prior to the emergence of shoots, consisted of culm. This observation was similar to previous reports of a preference for culm by captive and wild giant pandas during spring [9, 11, 14, 15]. An increase in the culm starch and hemicellulose matrix during the spring showed a distinct positive relationship with this increased culm consumption, while the fiber content during this same time frame showed a negative relationship. The mono- and polysaccharides in starch and hemicellulose likely become more bioavailable when culm fiber content is reduced. As very little fat is present in bamboo, and protein conversion to glucose is energetically costly, glucose in culm is a valuable calorie resource for giant pandas during spring. These observations improve our understanding of the nutritional drivers behind giant panda dietary preferences and underscores the need to study carbohydrate content (specifically starch, hemicellulose, and sugars) of bamboo in natural and captive habitats in relation to giant panda foraging and movement ecology.

This study also confirmed reports which suggested that the preference for leaves by giant pandas in the summer and fall was because leaves contained lower fiber content than culm, and more energy in the form of protein and fat. Here, we report that bamboo leaves also contained greater concentrations of free sugars than culm throughout the year, and add that the proportion of glucose, fructose and sucrose in bamboo leaves varied seasonally. Dierenfeld [8] noted that giant pandas meet their requirements for protein and energy through consumption of large quantities of bamboo each day. Nutritional balance for C and N, and the C/N ratio, therefore, may be met in large part by the ingestion of leaves that are available to giant pandas throughout the year. In addition, we suggest that giant pandas can display a seasonal feeding strategy to take advantage of nutrients available in culm during spring. Therefore, the requirement for maintaining an appropriate C/N ratio in spring may be met primarily through the consumption culm for some giant pandas at this time of the year.

The digestion coefficient for bamboo cellulose is low (9%) because giant pandas lack specialized compartments for extensive fermentation [4]. Hemicellulose that is made up of the carbohydrates glucose and xylose, however, are more bioavailable with digestion coefficients for giant pandas ranging 22 to 25% [4]. Wei et al. [18] suggested that the increase in hemicellulose content of bamboo leaves during summer was part of the signal for red pandas (*Ailurus fulgens*) to transition from foraging on culm and shoots to leaves. Here, we also reported that giant pandas consumed the greatest proportion of bamboo leaves during summer when leaf hemicelluloses were highest, and that giant pandas transitioned to culm when leaf hemicellulose content fell below 25%. Dierenfeld et al. [4] also reported that giant pandas refused bamboo with less than 25% hemicellulose content. The annual pattern for total bound sugars (of which ~60% was glucose) was similar to the annual pattern for hemicelluloses in both leaf and culm. Therefore, it follows that much of the measured bound glucose in bamboo culm is likely to be hemicelluloses. The linkages of glucose and xylose in hemicellulose may be broken down, in part, by stomach acids allowing for digestion and absorption in the lower GIT [4, 9]. The bacterial genes *β*-glucosidase and *β*-xylosidase also have been found in the giant panda GIT [19], and suggest that microbial digestion of the bound glucose and xylose making up hemicellulose may also occur. Therefore, giant pandas can gain valuable energy through absorption of these carbohydrates directly, as well as make use of the increased production of short chain fatty acids produced during microbial degradation.

Seasonal changes in nonstructural carbohydrates may also help to explain plant part and species preferences of giant pandas. Within plant cells, free sugars are photosynthetically produced to sustain plant growth. When photosynthetic capacity exceeds the plant’s needs, unused sugars are stored as starch, fructans, or sucrose in plastids and vacuoles of parenchyma cells [20]. This study reports that starch in leaves remained relatively constant throughout the year at ~ 2% DM (one outlier increased to approximately 14% starch) whereas culm exhibited an elevation in starch (overall species mean of 5.5%) during spring. The present study also found that AU and NU culms were greater in starch, and had lower fiber content compared to culms of AR and GL throughout most of the year. The fact that Memphis Zoo giant pandas preferred AU and NU bamboos compared to AR andGL, and selected for culm when starch was at its highest suggests that giant pandas may be seeking this easily digestible caloric resource. Changes in non-structural carbohydrates are thought to protect the plant during cold temperatures and thus are closely tied to phenological events [21]. Springtime chemical changes in bamboo plant tissues also have been attributed to mobilization of carbohydrates in preparation for new shoot emergence and rapid growth [22, 23]. Amylase in the small intestine breaks starch into glucose for absorption, and provides energy and precursors to lipid biosynthesis.

The ability to select plant parts with the greatest caloric value has been proposed to be a result of sweet perception by giant pandas, such that plant species or plant parts containing greater levels of bioavailable sugars are more desirable to the bear. As described above, the intake of culm in the overall diet of giant pandas increased within a month of the measured elevation of starch and bound glucose. In addition, giant pandas preferred leaves and culm when the proportion of free fructose in that plant part was highest. Nutritional theory states that animals regulate their intake of multiple nutrients to a nutritional objective that is dependent on their requirements for metabolism, growth, and reproduction at specific time points in their life history [24]. Therefore, animals must detect the composition of their diet in relation to their current and anticipated nutritional state. To accomplish this, sensory cells and receptors within the mouth and GIT detect key compounds including salts, sugars, and amino acids [24]. Giant pandas cannot distinguish “savory” flavors associated with proteinaceous foods due to the presence of a pseudogene in the place of normal genetic coding for the umami taste receptor [25]. Instead, Jiang et al. [26] surmised that the plant-based diet of giant pandas has led to a fully functional sweet perception that is lacking in meat eating carnivores. Experimental studies suggested that fructose and sucrose were potent sweeteners and strongly preferred by giant pandas even at low concentrations [26]. Therefore, the detection of sugars by giant pandas (and other plant consumers) has been hypothesized to motivate caloric consumption. The dietary preferences by giant pandas for select bamboo species and plant parts with corresponding elevated concentrations of carbohydrates further support this hypothesis.

Post-ingestive gastrointestinal discomfort can also result in learned associations to avoid specific diet items. Bamboo leaves are known to accumulate silica within plant cell walls to provide rigidity and act as a defense against plant foragers [8, 9, 27, 28]. Leaf ash, which has been reported to consist of 80% silica [29], was highest in leaves during winter and spring in the present study and may have further contributed to the giant pandas’ preference for culm. Schaller et al. [9] and Tabet et al. [30] also reported that silica concentrations in leaves were highest during the spring. In addition, volatile oils or cyanogenic compounds also can reduce the palatability of specific plant parts to foragers [31] and likely negatively influence foraging decisions by giant pandas. More research is needed to understand whether giant pandas make foraging decisions based on plant secondary metabolites or other plant compounds that have been reported to determine dietary selection in other selective and energy-restricted herbivores [32].

Declining nutrient value of bamboo leaves and their potential unpalatability during winter and spring poses a further restriction on the giant panda’s already specialized dietary niche. This study suggests that the dietary selection of spring culm allows giant pandas to take advantage of the greater biomass available in culm when it has the greatest concentration of starch and bound sugars, and reduced fiber content. Giant pandas remove the outer sheath from bamboo culm prior to eating, which has been described as a foraging strategy to select the most digestible portion of the culm [9]. The outer sheath layer of *P*. *pubescens* bamboo contained greater amounts of cellulose and lignin compared to the pith [33], and a similar pattern is suspected to exist for the *Phyllostachys* bamboos examined in this study. Culm samples in this study included both the pith and outer sheath. Consequently, culm fiber content reported here is likely much higher than what the bears were ingesting, and culm fiber content consumed is probably closer to leaf during spring. Schaller et al. [9] described passage rates for giant pandas of approximately 8 h for shoots, 10 h for culm and 14 h for leaves. Therefore, the preference of spring culm allows giant pandas to digest bioavailable carbohydrates, and pass the indigestible portions more quickly than if they were to consume leaves. The faster rate of food passage for culm versus leaf also limits the constraint to further forage consumption. This foraging strategy allows giant pandas to increase intake, allowing for a greater total volume of plant material (and corresponding nutrients) to pass through the digestive system daily. In the absence of more digestible and nutrient-rich bamboo shoots, culm is the next best dietary resource for giant pandas during spring. Because giant pandas do not have enlarged areas for fermentation, it makes sense that digesta passage rate is rapid.

Interestingly, the time of greatest culm consumption corresponds to the spring breeding period for the giant pandas. This is likely to be the most active time for giant pandas when energetic demands are at their highest and males search for mates. If all giant pandas are in search of culm and early shoots in spring, this also may have been the nutritional driver that led to the adaptation for seasonal breeding when these otherwise solitary animals aggregated in their search for the most nutritious food resources. Animals require many nutrients to sustain maintenance and growth at a given point in their life, as determined by the animal’s ability to detect its current needs. As such, some researchers have theorized that shoots and culm during the breeding season also contain specific estrogenic factors contributing to fertilization [8, 34]. Dietary selection patterns have also been described to be the result of giant pandas selection for calcium, phosphorus, or other minerals that may be limited at other times of the year [12]. During the time of this study (2008–2010), the body mass of the female at the Memphis Zoo was at its lowest during late winter and the greatest increase in body mass occurred when culm consumption was at its highest in spring. If similar fluctuations in body mass occur throughout the year for other captive and wild giant pandas, the consumption of more calorie-dense foods during spring may be essential for gaining sufficient body mass to maintain pregnancy. In other ursids, females maintaining a better body condition during the period of embryonic diapause were more likely to produce cubs in fall [35]. Bioavailable mono- and polysaccharides in bamboo culm and leaves are an important source of calories and undoubtedly contribute to the metabolic requirements for maintenance, growth, and reproduction for giant pandas.

## Materials and methods

### Bamboo collection, processing and nutritional analyses

Seven species of bamboo (*Phyllostachys* (*P*.*) aurea*, *P*. *aureosulcata*, *P*. *bissetii*, *P*. *glauca*, *P*. *nuda*, *P*. *rubromarginata*, *Pseudosasa (Ps*.*) japonica*) from established cultivars at the Memphis Zoo Bamboo Farm (Agricenter, Memphis, TN, USA) are regularly provided to the male and female giant panda to provide a diverse diet throughout the year. Four of these species of bamboo (*P*. *aurea*, *P*. *aureosulcata*, *P*. *glauca*, *P*. *nuda*) were selected for nutritional analysis as these species were of the same genus and preferred by giant pandas throughout the year (Kouba, personal observation). These four species are all endemic to China, and are a common diet staple for many giant pandas in the U.S. [7, 36]. Following harvest, bamboo was stored in a misted cooler for no more than 3 days prior to feeding. The animals are offered 2–10 kg bundles of bamboo at various intervals throughout the day (totaling 20–60 kg of bamboo offered). Bamboo were harvested from June 2008 through June 2010 during early morning hours (7–10 am). Mean culm diameter was 1.96, 2.10, 2.06, and 2.14 cm for *P*. *aurea* (AR), *P*. *aureosulcata* (AU), *P*. *glauca* (GL), and *P*. *nuda* (NU), respectively. Mean leaf length was 13.8, 9.84, 14.52, and 12.7 cm for AR, AU, GL, and NU, respectively. None of the cultivars were fertilized prior to- or during the course of nutritional analyses. Mean temperature at the cultivar during 2008–2010 was 16°C (range, 4–27) during Spring (March–May), 26°C (17–34) during Summer (June–September), and 8°C (-3-26) during Fall/ Winter (October–February). Annual precipitation each year ranged 132–182 cm / year. Rainfall during Spring, Summer, and Fall/ Winter months averaged 58, 40, and 61 cm, respectively (National Oceanic and Atmospheric Administration, National Centers for Environmental Information).

For each of the four bamboo species, three whole culms were selected each month from random locations (using a random number generator) within the stand, avoiding the first available row of bamboo to ensure sampled culms were at least 1-year-old. Leaves were stripped from branches and dried at 60°C for 24 hours. Bamboo culms were cut into 1–3 cm sections and dried for seven days. Branches were discarded since giant pandas typically do not consume this portion of the plant. After drying, samples were ground using a Wiley Mill Model 4 to pass through a 1 mm screen (Thomas Scientific, Swedesboro, NJ, USA).

Dried and ground samples were sent to the University of Illinois at Urbana-Champaign (Urbana, IL. USA) for analysis of ash, total dietary fiber (TDF), soluble dietary fiber (SDF), insoluble dietary fiber (IDF), acid detergent fiber (ADF), acid detergent lignin (ADL), crude protein (CP), total free sugars, and monosaccharides corrected for free sugars (bound sugars for the purpose of this study). Cumberland Valley Analytical Services (Hagerstown, MD, USA) performed the fat (ether extract; EE) and starch analyses.

Samples were dried at 100°C for 24 hours to determine dry matter (DM) content of the dried ground sample [37], and nutrient content was calculated in reference to total DM. Standard procedures for ash, CP, TDF, ADF, and ADL were performed as described previously [36]. Ether extract (EE), was measured using the AOAC Official Method [39]. Insoluble dietary fiber (IDF) was the remaining residue after enzymatic digestion of a subsample and subsequent filtration. The filtrate was precipitated with alcohol (SDFP). The remaining fraction that was not precipitable in alcohol was quantified by liquid chromatographic analysis and consisted largely of oligosacharrides (SDFS). Final SDF includes the sum SDFP and SDFS. Both IDF and SDF values were corrected for crude protein and ash [40]. TDF was the sum of IDF and SDF. The percentage of hemicellulose was calculated as the difference between the IDF and ADF.

Individual monosaccharaides (fructose, sucrose, arabinose, galactose, glucose, xylose, mannose, fucose, rhamnose) were extracted by acid hydrolysis and measured by anion-exchange high-performance liquid chromatography (HPLC) [41, 42]. The concentration of free (F) sugars (F-fructose, F-sucrose, F-arabinose, F-galactose, F-glucose, F-xylose, F-mannose, F-fucose, F-rhamnose, F-total) were measured through water extraction, filtration, and HPLC analyses of the resulting filtrate [43]. Free sugars were subtracted from the respective monosaccharide quantities to calculate ‘monosaccharides corrected for free sugars’, referred to as bound (B) monosaccharides (B-arabinose, B-galactose, B-glucose, B-xylose, B-mannose, B-total). Total starch content was determined by two-stage α-amylase, glucoamylase digestion of a subsample and measurement of absorbance at 510 nm [44].

### Foraging behavior

Annual changes in bamboo plant part selection by the male (Studbook number, 466) and female giant panda (Studbook number, 507) at the Memphis Zoo during 2008–2010 were recorded through live video observations as previously described [15, 16]. Both giant pandas are descendants of animals rescued from the Quinling Mountains, Shaanxi Province, China. The male and female were 13 and 11 years of age, respectively, at the beginning of the study period. Animals were monitored continually by veterinary staff and considered healthy (male body mass, 107 kg; female body mass, 90 kg). The behavioral ethogram focused on foraging patterns of the giant panda during the year that occurred while the animals consumed bamboo leaf and culm. Information on giant panda consumption patterns were collected in 20 min blocks by the method of instantaneous scan marking. The bamboo plant part consumed by each animal was categorized as leaf, culm, other, and unknown. Data collection sessions were initiated when the bear was observed to begin eating and data were recorded every 30 sec. Consumption patterns were averaged on a monthly basis. The Memphis Zoo is accredited by the Association of Zoos and Aquariums. All care and husbandry of giant pandas at the Memphis Zoo aligned with the guidelines determined by AZA’s Species Survival Plan for giant pandas. The Memphis Zoo granted permission for this study to be conducted by the authors under permits from the US Fish and Wildlife Service in support of scientific research of benefit to giant panda conservation. This study involved sampling of bamboo, and behavioral monitoring of giant panda feeding behavior through video recording without animal handling or diet manipulation. Therefore, this study was considered non-invasive and did not require an Institutional Animal Care and Use Committee protocol.

### Data analyses

The data presented are shown as the average for all species combined unless otherwise specified. All data were examined for normal distributions using the Shapiro–Wilks test prior to statistical analyses. A Kruskal-Wallis One Way ANOVA on Ranks with Dunn’s method for multiple comparisons was used to examine the annual nutrient differences among species and plant part. We also restricted nutrient comparisons by focusing on months where culm-eating and leaf-eating was highest. Thus, culm-eating season was restricted to March through May (culm was 80–95% of the overall diet) and leaf-eating season from July through September (leaf was 75–95% of the overall diet). Transitionary periods where mixed diets were shifting were excluded from this analysis. Species data were combined to examine differences in nutrient concentrations between plant parts and feeding seasons using a t-test or Mann-Whitney U test for parametric and nonparametric data, respectively. Sigma Plot 12.0 (Systat Software, Inc.) was used for both graphing and statistical analyses.
